# Motor coordination in school-aged children with different nutritional statuses following a non-immersive virtual reality intervention

**DOI:** 10.3389/fpubh.2026.1779788

**Published:** 2026-03-02

**Authors:** Ana Patrícia Da Silva Souza, Sandra Lopes De Souza, Maria Eduarda Rodrigues Alves Dos Santos, Ana Beatriz Januário Da Silva, Karollainy Gomes Da Silva, Robson Feliciano Da Silva, José Maurício Lucas Da Silva, Mayara Luclécia Da Silva, Érica Helena Alves Da Silva, Williclecia Walkiria Dias Ferreira, Paulo Roberto Leite De Arruda, Antonietta Cláudia Barbosa da Fonseca Carneiro, Waleska Maria Almeida Barros

**Affiliations:** 1 Postgraduate Program in Neuropsychiatry and Behavioral Sciences, Center of Medical Sciences, Federal University of Pernambuco, Recife, Brazil; 2 Department of Physiotherapy, Center of Health Sciences, FACOL University Center (UNIFACOL), Vitória de Santo Antão, Brazil; 3 Integrated Center of Neuroscience Technologies, FACOL University Center (UNIFACOL), Vitória de Santo Antão, Brazil; 4 Department of Medicine, Center of Health Sciences, FACOL University Center (UNIFACOL), Vitória de Santo Antão, Brazil

**Keywords:** children, motor coordination, nutritional status, physical activity, sedentary behavior, virtual reality

## Abstract

**Introduction:**

Children with low motor coordination tend to engage in less physical activity. They are more likely to gain weight, establishing a bidirectional relationship, as weight gain may further restrict their participation in physical activity. When opportunities for physical activity are enjoyable, the likelihood of children’s engagement increases, which may lead to higher levels of physical activity.

**Aim:**

To examine the effects of a non-immersive virtual reality game protocol on gross motor coordination in children with different nutritional statuses, and specifically, to compare sedentary behavior time between groups.

**Methods:**

In a quasi-experimental study, a non-immersive virtual reality game protocol was implemented for eight weeks in children aged 5 to 9 years from municipal public schools. Socioeconomic, anthropometric, motor coordination, and sedentary behavior time data were assessed.

**Results:**

Fifty-six children from five schools completed the study, with a mean age of 6.86 ± 1.51 years; 26 were girls (46.42%). Repeated-measures ANOVA showed no significant effect between groups (*p* = 0.149, η^2^p = 0.149). A negative Spearman correlation was observed between age and motor coordination at baseline (r = −0.627; *p* < 0.001) and at post-intervention assessment (r = −0.538; *p* < 0.001). Sedentary behavior time did not differ between groups (Welch’s ANOVA: *p* = 0.568).

**Conclusion:**

The intervention did not affect motor coordination. Younger children demonstrated better motor coordination, and sedentary behavior time was high and similar among the children. These findings highlight the importance of public health strategies that provide regular and adequate motor stimulation during childhood.

## 1 Introduction

The basic skills that enable children to perform simple movements and provide the foundation for normal development and the maturation of more complex abilities are referred to as Fundamental Movement Skills (FMS) (1). These skills can be categorized into locomotor skills (e.g., running, jumping, and galloping), object control skills (e.g., throwing, rolling, catching, and kicking), and postural control movements (e.g., trunk flexion and rotation) (2). Children are generally expected to master these skills by approximately seven years of age, when they begin to engage in more specialized skills associated with sports and exercise (3). Mastery of these movements through learning, practice, and reinforcement is essential for children’s independence and for the healthy development of physical, motor, and cognitive health (4).

Children with low motor coordination tend to engage in less physical activity and are more likely to gain weight, giving rise to a bidirectional relationship, as weight gain may restrict participation in physical activity (5, 6). Overweight and obesity have detrimental effects on the development of fundamental movement skills and on cognitive function, particularly during early childhood (7, 8). When opportunities for physical activity are enjoyable, the likelihood of children’s engagement increases, and, as a result, they tend to be more physically active (9). However, as children grow older, difficulties or impairments in performing basic locomotor and manipulative skills may limit opportunities for physical activity practice due to inadequate prerequisite development. Motor coordination, one of the most important abilities in early childhood, when properly developed, represents an important strategy for the long-term prevention of obesity, as well as for encouraging engagement in physical activity (10). Motor coordination plays a key role in the execution of physical and sports activities by influencing overall movement performance. Children involved in multisport activities exhibit better motor coordination compared with sedentary children (11). Moreover, school-aged children with normal weight demonstrate superior overall motor coordination, locomotor coordination, global object control, and manual control compared with their overweight or obese peers (12). Promoting and modifying health-related behaviors is easier in childhood than changing unhealthy habits in adulthood (13). Therefore, fostering the development of motor coordination may help promote healthier and more physically active lifestyles in children (14).

From this perspective, virtual reality (VR) games represent a combination of stimuli that influence brain morphofunctional aspects by increasing sensory, cognitive, motor, and social interaction stimulation (15). Among healthy children, VR is an effective means of improving certain domains of physical fitness, such as balance, postural stability, and agility (16). In children with autism, functional near-infrared spectroscopy revealed increased activation in the prefrontal cortex and parietal lobe, areas associated with executive control, attentional regulation, and sensorimotor integration. Additionally, increased premotor activity and activation of mirror neuron systems were observed, suggesting that virtual reality may facilitate learning through imitation and action observation, processes that are crucial for motor and social development (17). Moreover, VR increases the sense of enjoyment during physical activity and provides a distraction that helps children with overweight or obesity benefit from its effects (18). About motor coordination, VR has already been investigated in children with developmental coordination disorder (19), and autism (17), and in cross-sectional studies involving healthy children (20). However, no studies were identified that examined the effects of virtual reality on motor coordination among children with overweight/obesity, eutrophy, and thinness.

Considering that childhood overweight and obesity are associated with reduced levels of physical activity and gross motor coordination (21). It is necessary to understand whether strategies that encourage increased physical activity can enhance children’s gross motor coordination, regardless of nutritional status. Therefore, the present study aimed to examine the effects of a non-immersive VR (NIVR) game protocol on gross motor coordination in children with different nutritional statuses by comparing an experimental NIVR group with a control group and to specifically compare baseline sedentary behavior time between groups.

## 2 Materials and methods

### 2.1 Study design and participants

This was a quasi-experimental study with a sample consisting of children of both sexes, aged 5 to 9 years, enrolled in public schools in a municipality in northeastern Brazil. The five schools in which data collection and the intervention took place were randomized using the Research Randomizer program (Version 3.0). Children with a diagnosis of chronic diseases, such as arterial hypertension, diabetes mellitus, or heart disease, as well as those with physical or mental disabilities and/or a diagnosis of any disorder that limited the performance of the tests and procedures, were excluded from the sample.

The project was approved by the Human Research Ethics Committee of the Federal University of Pernambuco (CAAE: 58651822.8.0000.5208/approval number: 5.586.951). Data collection began in February 2023 and was completed in August 2025. All parents or legal guardians were informed about the study objectives, as well as its risks and benefits, and provided written informed consent. The children also signed an assent form.

#### 2.1.1 Procedures

Recruitment was conducted actively through informational meetings held at the school, with voluntary participation from caregivers. After consent was obtained, anthropometric assessments were performed to classify the children’s nutritional status. Participants were then allocated to groups by convenience, based on caregivers’ availability to bring the children to the sessions. Baseline comparability between groups was assessed, and relevant covariates (age, nutritional status) were controlled for in the analyses to reduce potential selection bias. The control group did not participate in any additional interventions during the study period, maintaining only their usual school activities. All variables were assessed before and after completion of the intervention period across the six groups. The pretest was conducted during the week preceding the start of the intervention, and the post-test during the week following its completion.

### 2.2 Measuring instruments

#### 2.2.1 Anthropometry

Children’s body weight was measured using a digital anthropometric scale (Welmy W200A LED, São Paulo, Brazil), with minimal clothing, barefoot, and without objects in their pockets, hands, or on their heads. Height was measured using a stadiometer (Welmy W200A LED, São Paulo, Brazil) with a total height of 220 cm and millimeter subdivisions. The child stood upright with the head positioned in the Frankfurt horizontal plane, arms hanging freely alongside the body, feet slightly apart, and heels in contact with the vertical surface of the stadiometer. The Anthro Plus software, version 2.0 (2007), provided by the World Health Organization (WHO), was used to calculate body mass index (BMI). Nutritional status categories—eutrophic, thinness, and overweight/obesity—were defined according to WHO reference standards (22).

#### 2.2.2 Gross motor coordination

A standardized test battery for children aged 5 to 14 years, developed in Germany, Körperkoordinationstest für Kinder (KTK), and validated for use in Brazil, was used (23). This test assesses several components of motor coordination and includes the following items:

- Balance: The child walks backward along a 3-m balance beam with decreasing widths (6, 5, and 4 cm). Scoring is based on the number of steps completed without losing balance.
- Hopping over an obstacle on one leg: The child is instructed to hop over a stack of foam squares using one foot at a time. After a successful hop with each foot, the height increases by 1 square (50 × 20 × 5 cm).
- Lateral jumping: The child performs consecutive jumps from side to side over a small slat (60 × 4 × 2 cm) as quickly as possible for 15 s.
- Platform shifting: The child begins standing with both feet on one platform (25 × 25 × 2 cm, supported at four points with a height of 3.7 cm each), places the second platform next to the first, and steps onto it; the first platform is then placed next to the second, and the child steps onto it. This sequence continues for 20 s.

Performance scores are summed and converted into a general Motor Quotient (MQ) and age-specific values. The general MQ classifies gross motor development into the following categories: “coordination insufficiency” (MQ 56–70), “coordination disorders” (MQ 71–85), “normal coordination” (MQ 86–115), “good coordination” (MQ 116–130), and “high coordination” (MQ 131–145).

#### 2.2.3 Sedentary behavior time

Children with poor motor coordination, as well as those who are overweight or obese, tend to engage less in physical activity and may spend more time in sedentary behaviors. Therefore, assessing baseline sedentary behavior and examining between-group differences may help clarify the study findings and inform the development of public health strategies. The South American Youth Cardiovascular and Environmental (SAYCARE) questionnaire was used. It has acceptable applicability and reliability for South American children and adolescents aged 3 to 18 years (24). For children, the questionnaire is completed by parents, whereas for adolescents, it is self-reported. Parents report the time their children spend in the following sedentary activities: watching television, using a computer, playing video games, and engaging in passive play, such as playing with toys or dolls, as well as painting activities. Sedentary behavior time is calculated as the sum of all reported activities (min/day) for weekdays and weekend days. Total daily sedentary behavior time is calculated by multiplying weekday sedentary time by five, adding weekend sedentary time multiplied by two, and dividing the result by seven.

### 2.3 Application of the non-immersive virtual reality game protocol

All study procedures were conducted at the school, either in the morning or afternoon, outside the children’s regular class hours. Virtual reality technologies are generally categorized as immersive and non-immersive. Immersive VR uses head-mounted devices equipped with sensors that capture users’ orientation and movements, allowing three-dimensional interaction and providing a sense of presence and sensory immersion. Non-immersive virtual reality (NIVR) uses conventional screens and motion sensors, enabling simultaneous interaction with virtual environments and the physical world (25).

In this study, the NIVR protocol consisted of a set of stimuli using Xbox 360 Kinect^®^ games, with sessions lasting 40 min, twice per week (on nonconsecutive days) for eight weeks. Children were instructed not to engage in any vigorous physical activity on the day before or on the day of any study procedure. The intervention included games that promoted movements such as lateral jumping, forward and backward displacements, upper limb elevation, squat jumps, and varied movement speeds, all aimed at guiding the performance of an avatar projected onto a wall using an EPSON^®^ PowerLite E20 projector. Each session was organized into three phases: warm-up, stretching, and NIVR game practice. Throughout the study period, participants exercised in pairs in a recreational, noncompetitive manner, under the supervision of a researcher.

For monitoring purposes, blood pressure, oxygen saturation, and heart rate (finger pulse oximeter) were assessed before the start and at the end of each session. The games used included Kinect Adventures (River Rush, Reflex Ridge, Space Pop, and Leaks), Kinect Sports (boxing, soccer, volleyball, tennis, baseball, and American football), and Just Dance, featuring various dance rhythms. Progression occurred through specific game modifications, with the addition of higher-complexity levels.

To facilitate familiarization with the NIVR games, all children participated in a preliminary introductory session before the start of the intervention. Before each session, children were instructed to remain within a demarcated area of the room, previously organized to ensure adequate motion capture by the equipment. The intervention protocol incorporated principles of motor learning, including use-dependent motor learning. The games required repetitive and variable execution of upper limb movements, such as shoulder abduction and adduction, rotations, and shoulder and elbow flexion and extension, as well as head movements, including rotation and lateral inclination. These movements are essential for spatial orientation, navigation, and visual perception and are involved in processes related to locomotion and balance maintenance. Lower limb movements included ankle dorsiflexion and extension, and knee and hip flexion and extension, contributing to dynamic stability and propulsion.

Principles of instructional motor learning were also applied. No specific instructions regarding movement execution were provided; children were only instructed to remain within the capture area, promoting spontaneous motor exploration. Game objectives were explained during the child’s first exposure, supporting task comprehension and cognitive engagement. Continuous visual and auditory feedback was provided through real-time avatar representation, allowing immediate motor adjustments during gameplay. Progression of game difficulty, combined with movement repetition and variability, supported ongoing adjustments in coordination, rhythm, and movement accuracy. In-game scoring and paired task execution were used to enhance engagement and adherence throughout the intervention period.

Regarding adherence, three consecutive absences resulted in exclusion from the study. After completion of the protocol and assessments, children in the control group were invited to participate in game sessions.

### 2.4 Sample size calculation

The G*Power software (version 3.1.9.4) was used, considering a repeated-measures analysis of variance between factors, with six groups and two measurements (*α* = 0.05; power = 0.80; *f* = 0.40; df = 5), which indicated a required sample of 48 participants.

### 2.5 Statistical analysis

All analyses were performed using Jamovi (version 2.7.6). Categorical variables were described as frequencies and compared using Fisher’s exact test. Age and BMI were presented as mean ± SD. Sedentary behavior time showed a normal distribution (Shapiro–Wilk, *p* > 0.05) and was compared using Welch’s ANOVA. The correlation between age and motor coordination was assessed using Spearman’s correlation coefficient (age not normally distributed, *p* < 0.05). Time effects (pre/post) and group effects were analyzed using repeated-measures ANOVA with age as a covariate. Effect size was estimated using partial eta squared (η^2^p) (*p* < 0.05). Assumptions of normality of residuals and homogeneity of variances were met (Shapiro–Wilk and Levene tests).

## 3 Results

**Figure 1:**
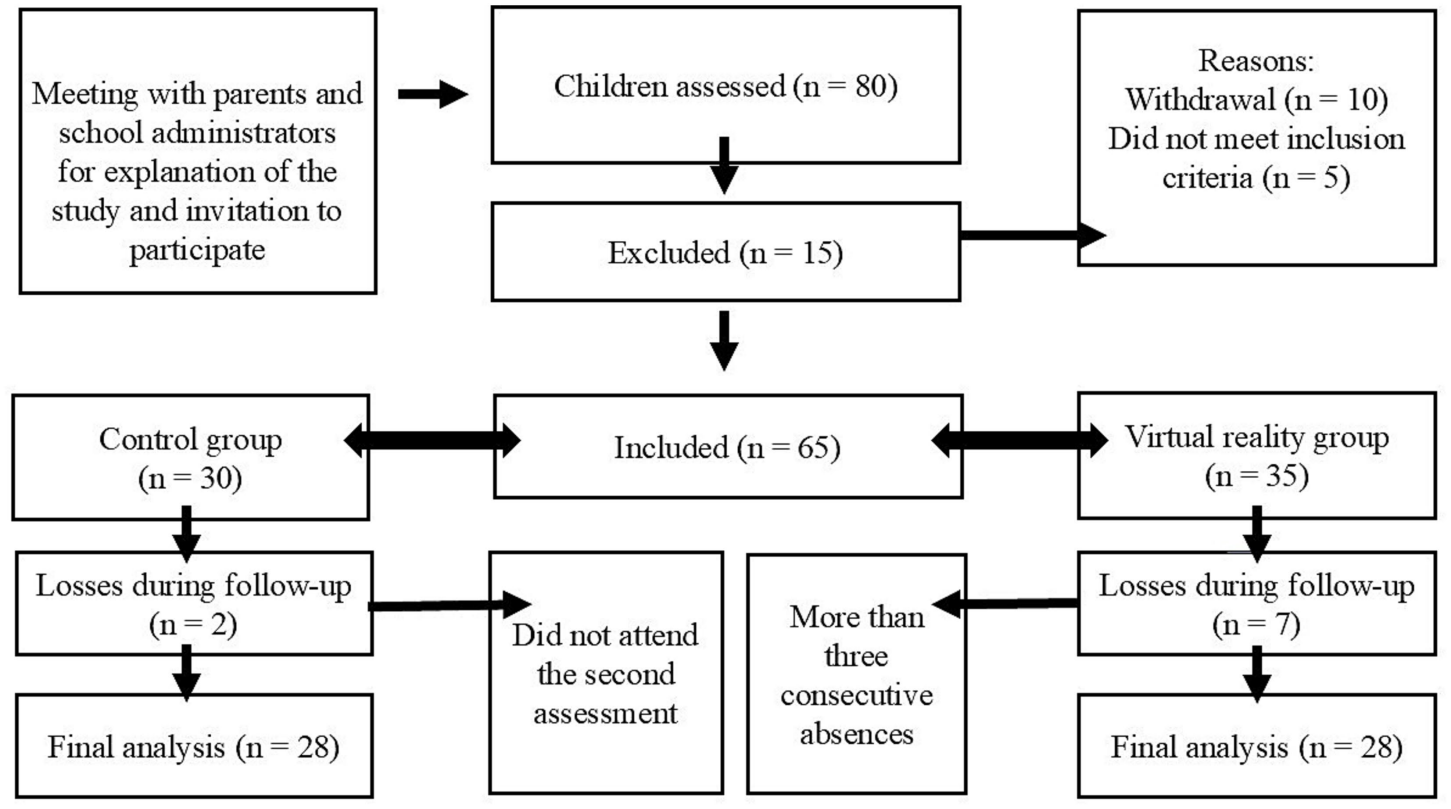
Flowchart of recruitment and participation in the study.

A total of 80 children from five municipal public schools were assessed; of these, 56 completed all stages of the study (Figure 1). The children reported no adverse events during the assessment process or NIVR sessions. Data collection procedures (baseline and post-intervention assessments and the intervention) were always conducted at the school and outside regular class hours.

### 3.1 Sample characteristics

The mean age of the 56 children was 6.86 ± 1.51 years; 26 were female, representing 46.42% of the sample. At baseline, data on socioeconomic status, anthropometric characteristics, and sedentary behavior time were assessed (Table 1). Allocation of children into the Control (C) and NIVR groups was performed according to nutritional status. Regarding parental education and income, mothers in the control group had completed elementary education and reported no monthly income, whereas mothers in the NIVR group had completed secondary education and reported a monthly income of up to one Brazilian minimum wage. No significant differences were observed between groups for sedentary behavior time (Welch’s ANOVA: F(5, 20.4) = 0.79; *p* = 0.568).

**Table 1:** Sample characteristics.

Variables	*n* (%)		*p*-value (Fisher’s exact test)
	Control (*n*)	NIVR (*n*)	
Sex			
Male	16 (28.57)	14 (25.00)	0.789
Female	12 (21.42)	14 (25.00)	
Total	**28**	**28**	
Age			
5	6 (10.71)	9 (16.07)	0.512
6	4 (7.14)	6 (10.71)	
7	5 (8.92)	6 (10.71)	
8	6 (10.71)	2 (3.57)	
9	7 (12.50)	5 (8.92)	
Total	28	28	
Ethnicity (self-reported)			
White	4 (7.14)	6 (10.71)	0.621
“Morena”	5 (8.92)	3 (5.35)	
Mixed-race (Parda)	16 (28.57)	13 (23.21)	
Black	0	1 (1.78)	
Not reported	3 (5.35)	5 (8.92)	
Total	**28**	**28**	
Number of people living in the household			
1 to 3 people	8 (14.28)	15 (26.78)	0.098
4 to 7 people	18 (32.14)	12 (21.42)	
Not reported	2 (3.57)	1 (1.78)	
Total	**28**	**28**	
Housing			
Owned	9 (16.07)	10 (17.85)	0.437
Rented	13 (23.21)	16 (28.57)	
Provided	4 (7.14)	1 (1.78)	
Not reported	2 (3.57)	1 (1.78)	
Total	**28**	**28**	
Maternal education level			
Elementary education	17 (30.35)	8 (14.28)	0.072
Completed secondary education	7 (12.50)	13 (23.21)	
Completed higher education	2 (3.57)	2 (3.57)	
Not reported	2 (3.57)	5 (8.92)	
Total	**28**	**28**	
Family income			
No income	14 (25.00)	6 (10.71)	**0.050**
Up to 1 minimum wage	9 (16.07)	17 (30.35)	
From 1 to 3 minimum wages	3 (5.35)	4 (7.14)	
Not reported	2 (3.57)	1 (1.78)	
Total	**28**	**28**	

Group (*n*)	Age (years)	BMI (Kg/m^2^)	SBT (minutes)
Groups by nutritional status and sedentary behavior time (SBT) in minutes			
Eutrophic C (10)	6.6 (±1.3)	15.1 (±0.90)	373 (±120)
Eutrophic NIVR (10)	6.4 (±1.7)	15.5 (±0.97)	460 (±116)
Thinness C (10)	7.1 (±1.7)	13.9 (±0.56)	354 (±169)
Thinness NIVR (10)	6.4 (±1.3)	13.4 (±0.53)	427 (±159)
Overweight/obesity C (8)	7.8 (±1.2)	20.6 (±2.55)	366 (±133)
Overweight/obesity NIVR (8)	7.0 (±1.4)	20.3 (±2.16)	383 (±97)

BMI, body mass index; NIVR, non-immersive virtual reality; C, control. Bold values indicate *n* = 28 per group; 0.050 indicates *p* ≤ 0.005.

### 3.2 Intervention effects

In the repeated-measures analysis of variance, the between-subjects effect indicated no statistically significant differences among groups (F(5, 49) = 1.71, *p* = 0.149) (Table 2). However, the large effect size (η^2^p = 0.149) suggests a trend toward variation between groups. A significant effect of age on gross motor coordination was observed (F(1, 49) = 23.79, *p* < 0.001, η^2^p = 0.327), indicating that performance was influenced by age regardless of nutritional status. In addition, a moderate negative Spearman correlation was identified between age and KTK scores at both baseline (r = −0.627; *p* < 0.001) and post-intervention after 8 weeks (r = −0.538; *p* < 0.001).

**Table 2:** Repeated-measures analysis of variance for the effects of time, group, and the time × group interaction on motor coordination, adjusted for age.

Effect	df	F	*p*	η^2^_p_
Within-subject effects				
Time	1	0.01	0.915	0.000
Time × Group	5	0.42	0.831	0.041
Time × Age	1	0.17	0.675	0.004
Between-subject effects				
Group	5	1.71	0.149	0.149
Age	1	23.79	<0.001	0.327

df – degrees of freedom; η^2^p – partial eta squared.

The estimated marginal means represent the adjusted values for each group, considering both time points (pre- and post-NIVR intervention) and controlling for the effect of age (Table 3). Although the difference was not statistically significant, a large effect size (η^2^p = 0.149) was observed between groups (Table 2). Higher adjusted mean values were observed in the NIVR groups compared to their respective control groups among eutrophic children (118 vs. 112) and overweight/obese children (111 vs. 104), while similar values were observed between the control and NIVR groups in the thinness category. The confidence intervals showed a large overlap between the groups.

**Table 3:** Estimated marginal means of group performance on the KTK gross motor coordination test.

Group			95% confidence interval	
	Mean (MQ)	Standard error	Lower limit	Upper limit
Thinness C	111	3.16	104	117
Thinness NIVR	110	3.19	103	116
Eutrophic C	112	3.17	105	118
Eutrophic NIVR	118	3.19	112	125
Overweight/Obesity C	104	3.66	97	112
Overweight/Obesity NIVR	111	3.53	103	118

MQ, motor quotient.

## 4 Discussion

The present study aimed to examine the effects of the NIVR game protocol on gross motor coordination in children with different nutritional statuses and, specifically, to compare sedentary behavior time. No significant effects of time or nutritional status were observed. However, age significantly influenced performance regardless of nutritional status, and a negative correlation was identified, indicating that gross motor coordination decreased with increasing age.

Although no statistically significant differences were found between groups, the large effect size indicated a trend toward better coordination in the intervention groups, particularly among eutrophic children and those with overweight/obesity. VR represents a means of encouraging these children to be more physically active. Interventions of this type may also benefit academic performance, and such initiatives can begin early in childhood. From this perspective, one study aimed to implement a 12-week exercise program and examine its impact on motor proficiency and cognitive skills in 71 preschool children with overweight and normal weight (26). Nutritional status was assessed using World Health Organization criteria, and motor skills were evaluated with the Test of Gross Motor Development (TGMD-2), which examines locomotor and object control skills. Cognitive function was assessed using the School Maturity Test. Regular physical exercise was found to promote positive effects on motor and cognitive skills in eutrophic and overweight preschool children.

Regarding children with thinness, one study examined how different domains of early childhood development were associated with nutritional status and included 627 mother–child pairs aged 12 to 36 months (27). Low body weight was associated with developmental delays, and these children were more likely to present greater delays in the gross motor domain compared with children of normal weight. The earlier attention is given to nutritional status, the greater the chances of benefiting children’s motor coordination.

Socioeconomic status (SES) should also be considered when examining motor coordination. In the present study, mothers of children in the control and intervention groups reported having no monthly income and an income of up to one Brazilian minimum wage, respectively, which may limit the purchase of nutritionally adequate foods. In this context, a study aimed to characterize motor proficiency in school-aged children and to investigate the association between chronic undernutrition and SES with motor proficiency (28). A total of 843 schoolchildren from Kolkata, aged 5 to 12 years, were assessed. Motor proficiency was evaluated using the Bruininks–Oseretsky Test of Motor Proficiency; nutritional status was determined according to World Health Organization reference standards; and SES was assessed using the updated Kuppuswamy scale. Severely undernourished children and those from lower SES backgrounds demonstrated motor proficiency levels below and well below average compared with normally nourished children and those from higher SES backgrounds. Thus, to enhance children’s gross motor coordination, it is not sufficient to promote increased physical stimulation alone; it is also necessary to provide opportunities to improve the nutritional conditions of this population.

The estimated marginal means of group coordination in the present study ranged from 104 to 118, corresponding to classifications between normal coordination and good coordination. However, age influenced performance across all nutritional statuses, showing that as children’s age increased, performance decreased. A study conducted in Brazil reported similar findings. Physical activity levels (measured by accelerometry over seven days) and motor coordination (TGMD-2 and KTK) were assessed among socially vulnerable youth, and the authors examined whether meeting physical activity guidelines was associated with motor coordination (29). A total of 1,017 individuals aged 3 to 14 years from three schools participated. Motor coordination levels were found to be low and also declined with increasing age in both sexes. The authors suggested that older children may have missed opportunities to develop motor coordination due to prolonged exposure to social vulnerability compared with younger children. It is therefore necessary to provide stimulating experiences and spaces for children throughout their growth and development, thereby increasing physical activity time. For children from lower SES backgrounds, the school may be the only setting in which such opportunities are available.

From a developmental perspective, motor behavior emerges from the interaction between neural maturation, social experiences, and environmental opportunities, as proposed by different models of motor development, including the Theory of Neuronal Group Selection, Dynamic Systems Theory, Ecological Systems Theory, and Sociocultural Theory (30). In this context, the sample of the present study included five-year-old children attending preschool. Early childhood education environments have been associated with higher levels of physical activity opportunities provided to children (31). Schools can therefore benefit children by prioritizing intentional practices that promote physical activity, including movement opportunities and the use of outdoor spaces, as strategies to increase physical activity levels and reduce sedentary behavior in young children (31, 32). In addition, having older siblings may provide younger children with more opportunities for learning through imitation and observation of actions, reinforcing motor development through socially mediated experiences (33). Approximately half of the children in the sample live in households composed of four to seven individuals, suggesting the presence of siblings, an aspect that warrants further investigation in future studies on motor coordination.

Within this framework, a recent meta-analysis showed that interventions lasting 60 min or longer, with a frequency of three or more sessions per week and a duration of 10 to 20 weeks, were effective in improving fundamental movement skills in children and that school-based interventions are effective in promoting these activities (34). However, physical activity levels have been declining during childhood and adolescence (29). In the present study, sedentary behavior time did not differ significantly between groups, and all groups presented mean values above 350 min per day. Although no specific time limit is established by the World Health Organization guidelines on physical activity and sedentary behavior, there is a recommendation to avoid prolonged sedentary time and to replace it with activities, even of light intensity (35). To intervene in a similar context, one study compared energy expenditure and intensity of active games with treadmill walking in a sample of 72 individuals aged 8 to 13 years (36). The games used were Kinect for Xbox 360, Adventure, Boxing I, Boxing II, and Dance. The authors found that these games elicited energy expenditure and moderate-intensity physical activity in both sexes and highlighted that VR in this modality may represent an interesting alternative for increasing physical activity levels in children and adolescents.

The use of VR with projection screens may enhance motor synchronization and attention under controlled conditions due to more direct visual processing. Such environments may improve motor performance by providing engaging feedback that supports learning and motor coordination (37). In addition, they offer a wider visual field with peripheral information, which may increase cognitive load (37). In the present study, task specificity was encouraged through the use of sports-based games, such as basketball, volleyball, boxing, and soccer. Previous research has shown that task-specific training using NIVR improves overall motor performance and balance in children aged 7 to 10 years with developmental coordination disorder (38). Although the intervention included relevant active ingredients, its impact may have been limited by the intervention duration, the dosage of practice, and contextual factors influencing motor coordination, such as time spent in sedentary behavior.

This study presented some limitations, such as the lack of assessment of daily physical activity levels and the inability to include all participating children in the same school shift. Consequently, the timing of assessments and the intervention was not the same for all children, which may have influenced performance as well as physical activity levels. Due to limited financial resources, it was not possible to use objective methods to assess sedentary behavior time, although accelerometry, a widely used and efficient research tool, would have been ideal. The study is subject to potential recall bias associated with self-report instruments, such as the questionnaire used. To mitigate this limitation, short recall periods and objective, structured questions were employed, and the questionnaire was validated for the study population. The intervention duration may also have been too short to demonstrate effects, particularly in children with high sedentary behavior time. Additionally, the heterogeneity of the sample and the number of groups analyzed may have reduced the statistical power to detect significant differences. The results are valid for this sample of children and should be interpreted with caution, as the study was not randomized. As a strength, the KTK was used, a motor skill test that is widely applied and recommended for research purposes. This study raised important questions and may serve as a reference for future research, taking its limitations into account.

Virtual reality is a feasible and accessible resource that attracts children’s interest and can be used as a strategy to increase engagement in physical activity, in addition to providing sensory, cognitive, and social stimulation for this population. However, it is important to promote education and awareness among parents, caregivers, public administrators, and teachers regarding the importance of reducing sedentary behavior time. There was no significant effect of the non-immersive virtual reality intervention on motor coordination. Sedentary behavior time was high and similar among children with different nutritional statuses. The findings highlight the need for public health policies with strategies that improve nutritional aspects and promote family, school, and leisure environments that provide regular and adequate motor stimulation, thus supporting the healthy development of the child.

## Data Availability

The original contributions presented in the study are included in the article/supplementary material, further inquiries can be directed to the corresponding author.
